# Biocontrol Ability and Action Mechanism of *Starmerella bacillaris* (Synonym *Candida zemplinina*) Isolated from Wine Musts against Gray Mold Disease Agent *Botrytis cinerea* on Grape and Their Effects on Alcoholic Fermentation

**DOI:** 10.3389/fmicb.2016.01249

**Published:** 2016-08-15

**Authors:** Wilson José Fernandes Lemos Junior, Barbara Bovo, Chiara Nadai, Giulia Crosato, Milena Carlot, Francesco Favaron, Alessio Giacomini, Viviana Corich

**Affiliations:** ^1^Department of Agronomy Food Natural Resources Animals and Environment, University of PadovaLegnaro, Italy; ^2^Interdepartmental Centre for Research in Viticulture and Enology, University of PadovaConegliano, Italy; ^3^Department of Land Environment Agriculture and Forestry, University of PadovaLegnaro, Italy

**Keywords:** antifungal activity, SAU-PCR, grape must, VOCs, lytic enzymes, fermentation, glicerol

## Abstract

Gray mold is one of the most important diseases of grapevine in temperate climates. This plant pathogen affects plant growth and reduces wine quality. The use of yeasts as biocontrol agents to apply in the vineyard have been investigated in recent years as an alternative to agrochemicals. In this work, fermenting musts obtained from overripe grape berries, therefore more susceptible to infection by fungal pathogens such as *Botrytis cinerea*, were considered for the selection of yeasts carrying antifungal activity. Thirty-six isolates were identified as *Starmerella bacillaris*, a species recently proven to be of enological interest. Among them 14 different strains were studied and antifungal activity against *B. cinerea* was demonstrated, for the first time, to be present in *S. bacillaris* species. The production of volatile organic compounds (VOCs), tested *in vitro*, was found to be the main responsible of *S. bacillaris* antifungal effects. All the strains were able to reduce *B. cinerea* decay on wounded grape berries artificially inoculated with gray mold. The colonization level of wound was very high reaching, after 5 days, a concentration of 10^6^ cells per ml of grape juice obtained after berry crushing. At this cell concentration *S. bacillaris* strains were used to ferment synthetic and natural musts. The sequential yeast inoculation, performed by adding *S. cerevisiae* 48 h after *S. bacillaris*, was needed to complete sugar consumption and determined a significant increase in glicerol content and a reduction of ethanol and acetic acid concentrations. The high wound colonization ability, found in this work, together with the propensity to colonize grape berry and the interesting enological traits possessed by the selected *S. bacillaris* strains allow the use of this yeast as biocontrol agent on vine and grape berries with possible positive effects on must fermentation, although the presence of *S. cerevisiae* is needed to complete the fermentation process. This work introduces new possibilities in wine yeast selection programs in order to identify innovative wine yeasts that are simultaneously antifungal agents in vineyards and alternative wine starters for grape must fermentation and open new perspective to a more integrated strategy for increasing wine quality.

## Introduction

*Botrytis cinerea* is one of the most important fungal plant pathogen that causes serious gray mold disease in more than 200 economically relevant plant species during pre-harvest (especially when plants are grown under protection), and post-harvest (Agrios, 2005). Grapes, vegetables, berries, and stone fruits cultivated worldwide are the most susceptible to this fungal disease (Rosslenbroich and Stuebler, 2000). The fungal agent infects leaves, stems, flowers and fruits of plants, either by direct penetration or through wounds caused by cultivation practices. This fungus kills host cells through the production of cell wall degrading enzymes, phytotoxic metabolites and reactive oxygen species accumulating after the induction of a plant-produced oxidative burst. Thanks to degrading enzymes, *B. cinerea* succeeds in the decomposition and consumption of different plant tissues (van Kan, 2006). Infestation is stimulated by high humidity, particularly if free moisture is present on the plant surface and low temperatures (Williamson et al., 2007). Generally the control of the disease is achieved by the use of synthetic fungicides (Elad and Evensen, 1995). From the middle of the 1990s, fungicides with excellent activity against *B. cinerea* came to the market and more recently the control of the disease was mainly achieved by integrating several cultural methods with the use of these fungicides (Rosslenbroich and Stuebler, 2000). Although, synthetic fungicides are effective, their continued or repeated application has disrupted biological control by natural enemies of the fungus and stimulated the development of resistant pathogen populations, leading to widespread outbreaks of the disease (Elad et al., 1992). The increasing concern over the adverse agronomical and environmental effects of synthetic fungicides brought to search new types of crop protection methods without or with reduced use of conventional fungicides. The salts of weak acids, such as sodium benzoate and potassium sorbate, can inhibit growth of several post-harvest fungal pathogens. These compounds present several benefits as they possess low toxicity toward mammalians, a wide spectrum of activity and are relatively cheap. However, these compounds need to be used at concentrations that can determine potential organoleptic changes of the products. For example, calcium propionate completely inhibited mycelial growth of *B. cinerea* at a concentration of 5% (w/v) (Droby et al., 2002). Essential oils obtained from aromatic and medicinal plant species have been proposed as new classes of possibly disease control agents, since they are a rich source of bioactive chemicals. These chemicals are often active against a limited number of species, are biodegradable to nontoxic products and are potentially suitable for integrated use (da Cruz Cabral et al., 2013). Specific activity against *B. cinerea* was found in essential oils obtained from the aerial parts of aromatic plants, which belong to the *Lamiacea* family, such as origanum (*Origanum syriacum* L. var. *bevanii*), lavender (*Lavandula stoechas* L. var. *stoechas*), and rosemary (*Rosmarinus officinalis* L.; Soylu et al., 2010). Traditional medical plants from Africa and Asia were found to be a source of essential oils proposed for post-harvest control of gray mold (Tripathi et al., 2008). Among the alternatives to synthetic fungicides, the use of plant resistance inducers demonstrated the potential for large-scale application. The induced resistance can be defined as an increased expression of natural defense mechanisms of plants against different pathogens provoked by external factors of various type: elicitors of pathogenic origin (glucans, proteins, lipids, etc.); abiotic elicitors, including synthetic harmless chemical products (Edreva, 2004). Some molecules, that act as inducers, also present antimicrobial activity. That is the case of chitosan that decreases gray mold and other fungal diseases through the reduction of mycelial growth and spore germination and induction of morphological alterations in the fungal pathogen. Moreover, chitosan acts as a potent elicitor to enhance plant resistance (Amborabé et al., 2008; El Hadrami et al., 2010). An alternative strategy to reduce gray mold disease is based on the selection and application of biocontrol agents. Among the microorganisms used as biocontrol agents, yeasts have been targeted by many surveys as potential mold antagonists, focusing mainly on post-harvest diseases, since they are naturally occurring on fruits and vegetables, and have shown great ability to colonize wound sites (Bai et al., 2008). Some have been present on the market for a long time and showed specific activity against *B. cinerea*. *Candida oleophila*, the base of the commercial product “Aspire,” is recommended for the control of post-harvest decay in citrus and pome fruits. Its modes of action include nutrient competition, site exclusion, and direct mycoparasitism (Droby et al., 2002). The yeast *Cryptococcus albidus*, included in the commercial product “Yield Plus,” is an antagonist isolated from peach fruit and effective against the pathogen *B. cinerea*in apple (Fan and Tian, 2001). As regards other yeast species the investigations as biocontrol agents are still ongoing. Recently, the ascosporic yeast *Metschnikowia fructicola* AL27 was tested on several apple varieties and found to be as competitive as the chemical fungicides used as control (Spadaro et al., 2013).

Focusing on viticulture, gray mold is one of the most important diseases of grapevine in temperate climates worldwide and can cause extensive economic losses through grape desiccation, rot and biochemical changes that reduce wine quality. Biological control of *B. cinerea* is a successful strategy that has been introduced as an alternative to synthetic fungicides in grapevine cultivation. Filamentous fungi from the genera *Trichoderma, Ulocladium*, and *Gliocladium*, bacteria from the genera *Bacillus* and *Pseudomonas* and, lately, yeasts from the genera *Pichia* and *Candida* have been used as biocontrol agents (Jacometti et al., 2010). Recently, an integrated approach that combined low dosage of fungicides and antifungal yeasts has been tested in order to reduce chemicals concentration and enhance biocontrol efficacy. *Hanseniaspora uvarum* was tested under laboratory conditions in combined treatment with NH_4_–Mo, showing inhibitory effects on spore germination and mycelial growth of *B. cinerea in vitro* and induced defense reactions in grape berries (Liu et al., 2010). Although several yeasts with antifungal property have been successfully identified, yeast selection to find out new biocontrol agents remains challenging, and species—to—species interaction studies are of great interest to understand native and introduced fungal population dynamics in both vineyard and cellar. Indeed, after grape harvest, antifungal yeasts become part of the must microbiota and, if well adapted to must condition, they could have a role during the fermentation process and therefore directly influence wine quality. At the moment, no information are available about the fate of selected yeasts proposed as biocontrol agents during must fermentation and winemaking, although they can be found on the grape surface at high level due to repeated treatments. Moreover, the possibility to select yeasts that are simultaneously antifungal agents in vineyards and wine starters for grape must fermentation is completely unexplored.

Non-*Saccharomyces* yeasts are a group of wine-related yeast species once defined spoilage microorganism. Generally they are well adapted to vineyard condition and are predominant in grape musts during the early stages of fermentation. Recently, there has been a re-evaluation of the role of these yeasts, as some of them were found to enhance the analytical composition and aroma profile of the wine (Ciani and Comitini, 2015).

*Starmerella bacillaris* (synonym *Candida zemplinina*) is a non-*Saccharomyces* yeast, commonly found on grapes and particularly associated with botrytized grapes and wines fermented from these grapes (Csoma and Sipiczki, 2008; Magyar and Tóth, 2011; Duarte et al., 2012; Masneuf-pomarede et al., 2015; Wang et al., 2015). Magyar and Tóth (2011) investigated the technological properties of *C. zemplinina* strains evidencing an extremely poor ethanol yield from sugar consumption, high glicerol and moderate volatile acids production. High glicerol production contributes to palate fullness (“body”) of wine, whereas high acetic acid content confers an unpleasant vinegar aroma. Therefore, with the aim of improving wine quality, *S. bacillaris* was recently tested, together with *Saccharomyces cerevisiae*, in sequential and mixed yeast inoculations during grape must fermentation to balance the glucophilic character of the *Saccharomyces* species, to increase glycerol concentration in wine and, due to the low ethanol yield, to reduce ethanol content (Rantsiou et al., 2012; Bely et al., 2013; Wang et al., 2014).

With the aim to investigate the double role of *S. bacillaris* as both potential biocontrol agent and unconventional enological starter, 14 strains belonging to this species were studied in this work to evaluate their antifungal activity against *B. cinerea*, both *in vitro* and *in vivo*. Moreover, the technological properties of these non-*Saccharomyces* strains were evidenced at lab-scale, both in single-strain fermentation and in sequential fermentation together with *Saccharomyces cerevisiae*.

## Materials and methods

### Isolation and characterization of yeast isolates

The yeast strains used in this work were isolated from fermenting musts obtained from dried grape of Raboso piave variety. They were collected during two harvests in two wineries located in the winemaking area of Appellation of Origin Bagnoli (North-East of Italy) where the production of Friularo Bagnoli Passito wine is performed. A total of 360 yeast colonies were isolated on WL agar medium (Oxoid) plates. All yeasts were identified at species level by PCR-RFLP analysis of the ITS1-5.8S-ITS2 rDNA region and D1/D2 region sequence analyses as described by Bovo et al. (2011). A BLAST search on sequence results gave the most probable species identification. Thirty-six isolates identified as *S. bacillaris* were characterized at molecular level using SAU-PCR method, as described by Corich et al. (2005). SAU-PCR amplification patterns were analyzed using the software GelComparII V. 3.5 (AppliedMaths).

### Extracellular lytic enzymes activity

*S. bacillaris* strains were screened for the production of extracellular cellulase, xylanase, lipase, pectinase and proteinase using plate tests as described by Lorenzo et al. (2013). The presence of extracellular chitinolytic activity was tested on glycol chitin agar medium (yeast nitrogen base, 6.7 g/L, glycol chitin, 5 g/L, agar 16 g/L). After the growth of the yeast colonies a solution containing 500 mMTris-HCl pH 8.9 with 0.01% w/v of Calcofluor white MR2 was poured on the plates. The plates were incubated for 10 min. Subsequently, the solution was discarded and replaced with water overnight. The presence of chitinolytic activity was evidenced by the observation of dark lytic plaques, where the colonies were present, on a light background under UV exposure. Extracellular β-glucosidase activity was evaluated using the esculin (esculetin 6-O-glucoside) agar hydrolysis test described by Njokweni et al. (2012) on Esculin agar (esculin 1 g/L, YNB 1.7 g/L, 0.5 g/L ferric citrate, agar 16 g/L) plates. Extracellular β-glucosidase activity were also tested by evaluating yeast growth on Cellobiose agar (cellobiose 5 g/L, YNB 6.7 g/L, agar 16 g/L) plates after incubation at 30°C for 72 h.

### *In vitro* antagonistic activity

The antagonistic activity on agar plates and volatile organic compounds (VOCs) assay was performed as described by Parafati et al. (2015), and modified as follows. The *B. cinerea* strain used was BC0510.

#### Antagonistic activity on agar plates

The yeast and mold strains to be tested were, respectively, growth on YPD for 24 h and on PDA for 5 days at 25°C. Each yeast strain was streaked orthogonally from the center of a plate, containing PDA (Potato Dextrose Agar) medium at two different pH (5.5 and 3.5). Simultaneously, for each plate 2 mycelial discs (6 mm square plug) of *B. cinerea* were placed on agar plates 3 cm away from the yeast streak. A control plate was prepared inoculating only *B. cinerea*. At the end of the incubation period (5 days at 25°C) the radial growth reduction was calculated in relation to the growth of the control as follows: %I = (C − T/C)^*^100, where %I represented the inhibition of the radial mycelial growth, C was the radial growth measurement in control and T was the radial growth of the pathogen in the presence of yeast strains. The assay was performed using four replicates for each yeast strain and pH.

#### Effects of volatile organic compounds (VOCs)

A dual culture method was used to evaluate the efficacy of volatile compounds produced by yeasts against *B. cinerea*. Aliquots of 20 μL of yeast suspensions (10^7^ cells/mL) were seeded on plates with PDA at two pH values, 5.5 and 3.5, and incubated 4 days at 25°C. Aliquots (10 μl) of the conidial suspension of *B. cinerea* (10^6^ conidia/mL) were inoculated on PDA and dried at room temperature. The plates with *B. cinerea* conidia were individually covered face to face under the plates containing the yeast strains. The controls were prepared facing the plates containing *B. cinerea* suspension with un-seeded PDA plates. Each plate pair was wrapped with two layers of Parafilm around the edges to prevent air leakage, and incubated a 25°C. The radial growth reduction of *B. cinerea* was calculated after 5 days of incubation as previously described.

#### *In vivo* antagonistic activity

In order to assess the efficiency of yeasts as biocontrol agents, the method described by Parafati et al. (2015), with slight modifications, was used. Table grape fruits derived from orchards located in Padova, Italy. Healthy and homogeneous grape berries were selected, washed and surface-disinfected. Artificial wounds were performed and inoculated with 10 μL drop of 10^6^ conidia/mL of *B. cinerea*. After air drying (2 h), a 10 μL drop of 10^8^ cells/mL of yeast were added to each wound. The same amount of 0.09% NaCl buffer (20 μL) was used in the control. For each strain 10 grape berries were used. The grape berries were placed on plastic packaging trays. To create a humid environment, a wet paper was placed on cavity trays coated with a plastic bag. The trays were incubated at 25°C and 95% relative humidity (RH) for 5 days after inoculation to provide favorable conditions for the disease development. The disease severity (DS) was evaluated by using an empirical 1-to-4 rating scale evaluating both soft rot size and mycelium growth: + barely visible symptoms, ++ small, +++ intermediate, ++++ large (comparable to control).

Data concerning the disease reduction incidence (DRI) was calculated as follow (DRI) = (C − T/C)^*^100, where C was the average radial growth measurement in control (10 berries), and T was the radial growth of the pathogen in the presence of yeast strain in each berry.

The lesion diameter (LD) was evaluated by measuring the average diameter of the damaged area 5 days after *Botrytis* inoculation. Each yeast strain was tested on 10 berries.

### Fermentation trials in synthetic and natural must

#### Inoculum preparation

A loopful of a 3-days-old culture of each yeast strains from YPD agar plate (yeast extract 10 g/L, peptone 10 g/L, dextrose 20 g/L) was used to inoculate 10 mL of YPD broth in 50 ml tubes. A stationary phase culture with approximately 10^7^–10^8^ cells/mL, determined by OD measurements and confirmed by means of plate counts analysis (CFU/ml), was obtained after 24 h of incubation at 30°C. In single-strain fermentation the inoculum concentration was 2 × 10^6^ cells/ml. In sequential fermentation the same inoculum size for both *S. bacillaris* strain and *S. cerevisiae* EC1118 (1–1.5 × 10^6^ cells/ml) was used. EC1118 was added 48 h after the inoculum of *S. bacillaris*.

#### Fermentation conditions

Fermentations were run in synthetic and natural musts. The synthetic must MS300 was prepared as described by Bely et al. (1990) with the addition of 100 g/L of glucose, 100 g/L of fructose and 6 g/L of malic acid, pH 3. Incrocio Manzoni grape must, containing 160 g/L of reducing sugars (pH 3.5) was used. In the fermentation trials 120 ml capacity bottles fitted with closures that enabled the carbon dioxide to escape and containing 100 ml of must were used. After yeast inoculation the bottles were incubated at 25°C. The fermentation process was followed by measuring twice a day the weight loss of each culture. When the weight loss was lower than 0.05 g per day the fermentations were considered concluded. All the fermentation trials were performed in triplicate.

### HPLC analysis

Ethanol, glicerol, fructose and glucose concentrations were quantified with HPLC (Shimadzu, Japan) equipped with a refractive index detector, set at 600 nm wavelength, while for the acetic acid quantification a UV detector was used.

The concentrations, expressed as g/L, were calculated by using calibration curves of the individual compounds. The chromatographic conditions were realized with the ROA-Organic Acid H+ column (Phenomenex, USA), which was run at 65°C with 5 mM H_2_SO_4_ as the mobile phase, with a flow rate of 0.5 mL/min.

### Statistical analysis

The statistical data analysis was performed with XLSTAT software, vers.7.5.2 (Addinsoft, Paris, France) using the principal component analysis (PCA) and the one-way analysis of variance (ANOVA) at 95% accuracy level. Fisher's test was used as comparison test when samples were significantly different after ANOVA (*p* < 0.05).

## Results

### Isolation, identification, and evaluation of extracellular activity of yeast isolates

A total of 360 yeast colonies were isolated from fermenting musts for the production of Friularopassito wine. By means of PCR-RFLP analysis of the ITS1-5.8S-ITS2 rDNA region and D1/D2 rDNAregion sequencing (Kurtzman and Robnett, 1998) each isolate was identified at species level. A total of 36 isolates were identified as *Starmerella bacillaris*. The characterization at strain level, by means of Sau-PCR (Corich et al., 2005) and the cluster analysis of the amplification pattern (see Supplementary Material SM_1), allowed the selection of 14 different strains listed in Table 1. All the strains were tested for the production of extra-cellular enzymes using specific plate assays. Some of the activities are of industrial interest: beta-glucosidase, cellulase, lipase, and xylanase. Pectinase, protease, chitinase are involved in degrading mold cell wall. The results of the screening are reported in Supplementary Material SM_2. All the strains showed chitinase activity, although at low level. Only strains FRI719 and FRI751 produced proteolytic enzymes as they were able to grow on skin milk. None of the other activities tested was found in any strain.

**Table 1:** **Yeast strains used in this work**.

**Strain**	**Species**	**Origin**
FRI719	*S. bacillaris*	Winery A
FRI728	*S. bacillaris*	Winery A
FRI729	*S. bacillaris*	Winery A
FRI751	*S. bacillaris*	Winery A
FRI754	*S. bacillaris*	Winery A
FRI779	*S. bacillaris*	Winery A
FRI7100	*S. bacillaris*	Winery A
PAS13	*S. bacillaris*	Winery B
PAS55	*S. bacillaris*	Winery B
PAS66	*S. bacillaris*	Winery B
PAS92	*S. bacillaris*	Winery B
PAS103	*S. bacillaris*	Winery B
PAS151	*S. bacillaris*	Winery B
PAS173	*S. bacillaris*	Winery B
EC1118	*S. cerevisiae*	Industrial strain

### *In vitro* antagonistic activity

**Figure 1:**
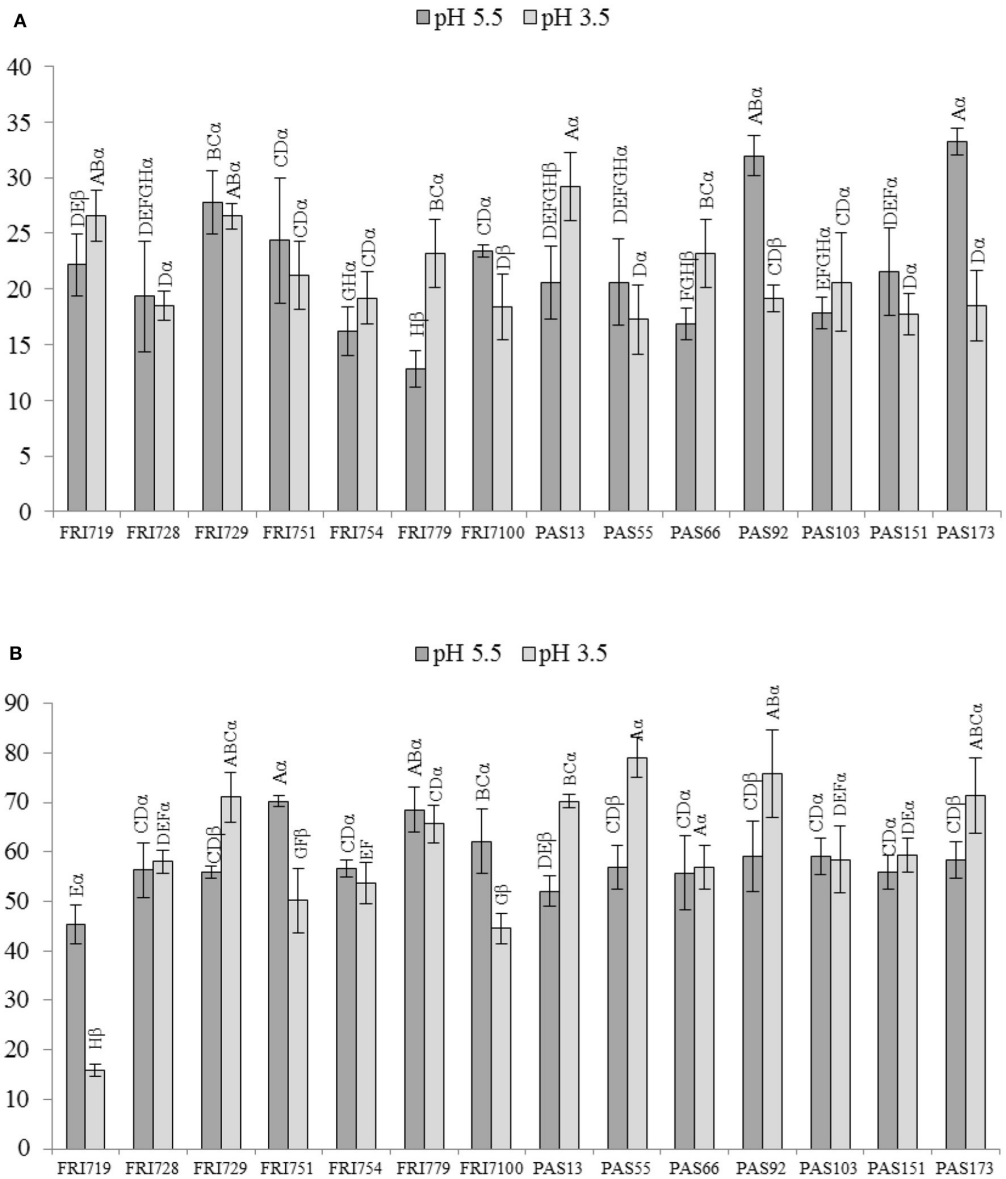
*****In vitro*** antagonistic activity of ***S. bacillaris*** strains against ***B. cinerea*** on PDA plate at pH 5.5 and 3.5**. Growth inhibition, measured as inhibition percentage of the radial mycelial growth, induced by yeast cells **(A)** and by volatile organic compounds (VOCs) **(B)**. Vertical bars indicate the standard error of the mean. Statistical analysis: one-factor ANOVA (*p* < 0.05). At the same pH, mean values followed by the same Roman letter are not significantly different according to Fisher's test (*p* ≤ 0.05). For each strain mean values obtained at pH 5.5 and 3.5, and followed by the same Greek letter are not significantly different according to Fisher's test (*p* ≤ 0.05).

Data from dual culture assays are reported in Figure 1. All the *S. bacillaris* strains were able to inhibit the growth of *B. cinerea* mycelium both at pH 5.5 and 3.5 when co-cultivated with *B. cinerea* (Figure 1A). The percentage of the inhibition of the radial mycelial growth ranged from 12 to 33. Strains FRI719, FRI779, PAS13, PAS66 showed higher antagonistic activity at pH 3.5 than at pH 5.5. On the contrary FRI100, PAS92 and PAS173 showed higher antagonistic activity at pH 5.5. In the other cases no significant differences were detected. In these conditions, for all the strains, the inhibition level found was limited, although comparable with that found in literature for some other yeast species (Parafati et al., 2015). This could be due to the different growth rate of *S. bacillaris* and *B. cinerea* on PDA medium where *S. bacillaris* can not found the optimal growth conditions. As the two microorganisms were inoculated simultaneously, *S. bacillaris* inhibited, only partially, the fungal growth.

The inhibition of *B. cinerea* mycelium growth due to the production of volatile compounds by *S. bacillaris* strains was tested, as well (Figure 1B). To overcome the different growth rate between the two microorganisms, plates inoculated with *S. bacillaris* strains were incubated 4 days at 25°C before covering face to face each plate with that containing *B. cinerea*. Generally, results showed notably higher inhibition percentages than those found when *B. cinerea* and each yeast strains were co-cultivated on the same plate: the values ranged from 44 up to 79%. Comparing the results with those of the previous growth inhibition assay, only PAS13 and FRI100 confirmed their inhibition ability in relation to the pH of the growing medium.

### *In vivo* antagonistic activity

**Figure 2:**
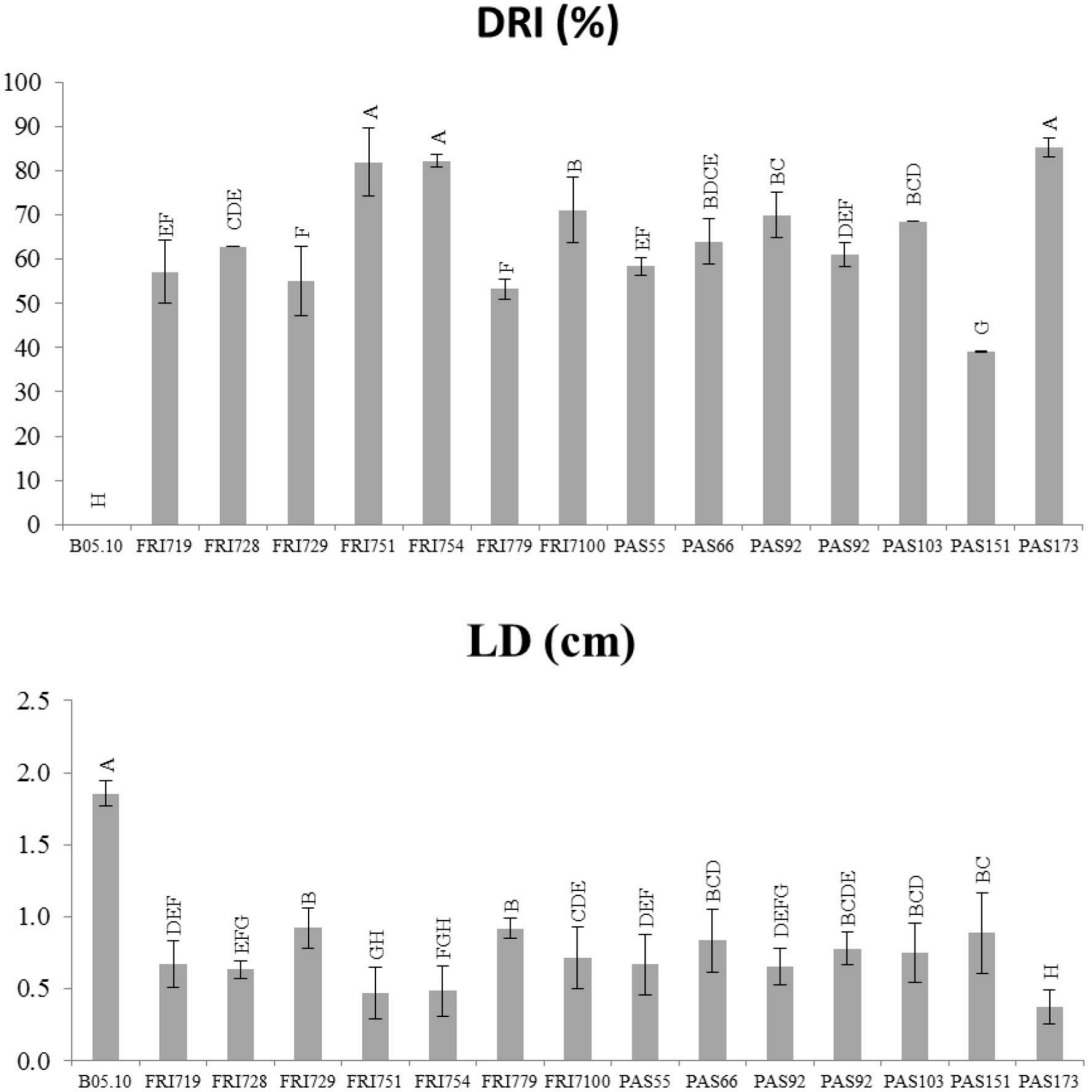
*****In vivo*** antagonistic activity of ***S. bacillaris*** yeast strains in inhibiting gray mold decay on grape berries**. Effect of yeasts is referred to disease reduction inhibition percentage (DRI%), and lesion diameter (LD) caused by *Botrytis cinerea* 5 days after incubation at 25°C. Vertical bars indicate the standard error of the mean. Mean values followed by the same letter are not significantly different according to Fisher's test (*p* ≤ 0.05).

A qualitative evaluation of the efficacy of the tested yeasts in reducing gray mold growth on grape berries is reported in Table 2. Although at different levels, all yeasts decreased the size of decay (soft-rot developed area) and the mycelium growth. Eight strains out of 14 showed remarkable effects on the developing of the *Botrytis* infection. In details (Figure 2), the DRI values ranged from 39 up to 85%. Strains FRI751, FRI754, PAS173 showed the highest gray mold decay as their values were significantly higher (*p* < 0.05) than those found for the other strains. The LD evaluation confirmed the remarkable ability of *S. bacillaris* strains to reduce the infection size, although only small differences were found between strains. When *S. bacillaris* strains were present in the wound the LD was always lower than 1 cm, while for the control the LD size was 1.8 cm. Strain PAS173 showed the highest level of LD reduction that was significantly different (*p* < 0.05) from those found for the other strains. At the end of the incubation time (5 days) each grape berry was squeezed and homogenized and *S. bacillaris* concentration was determined by plate counts (Table 2). The yeast concentration was very similar for all the strains ranging from 1.83 × 10^6^ up to 2.35 × 10^6^ CFU/ml.

**Table 2:** **Qualitative evaluation of the ***Botrytis*** infection severity on grape berries and yeast cell concentration in the grape juice obtained by berry squeeze after 5 days from yeast inoculation**.

**Strain**	**Soft rot**	**Mycelium**	**10^6^ CFU/mL**
Control	++++	++++	−
FRI719	+	+	1.98 ± 0.07
FRI728	+	+	2.07 ± 0.09
FRI729	++	+	1.83 ± 0.08
FRI751	+	+	1.89 ± 0.10
FRI754	+	+	2.35 ± 0.01
FRI779	++	+	2.10 ± 0.07
FRI7100	+	+	2.20 ± 0.04
PAS13	++	+	2.25 ± 0.08
PAS55	++	++	2.05 ± 0.05
PAS66	++	++	2.08 ± 0.08
PAS103	++	++	2.38 ± 0.11
PAS151	+	++	2.04 ± 0.07
PAS92	+	+	2.07 ± 0.10
PAS173	+	+	2.34 ± 0.06

### Fermentation activity in synthetic must

The fermentation activity of the 14 strains of *S. bacillaris* were evaluated in synthetic must MS300 at 20°C using an inoculum of 2 × 10^6^ cells/ml. The CO_2_ production was followed during all the fermentation process. To assess strain fermentation performances, the fermentation vigor, in terms of CO_2_ production after 48 h of incubation, was considered in order to evaluate the adaptation ability to the must conditions. CO_2_ production after 312 h was considered, as at these fermentation step the widest range of CO_2_ production was found between strains. The industrial wine strain *S. cerevisiae* EC1118 was used as control. The fermentations of *S. bacillaris* strains were stopped after 624 h when the fermentation of *S. cerevisiae* EC1118 was completed. As expected, *S. bacillaris* strains showed a very low CO_2_ production if compared to that of EC1118 (see Supplementary Material SM_3). Fermentation performances were very similar between strains as no significant differences were found after 312 and 624 h of incubation. Regarding fermentation vigor, strains FRI719, FRI728, and PAS92 showed a significant delay in the fermentation start (0.03, 0.01, 0.07 g/100 mL CO_2_ after 48 h, respectively). Strain PAS173 showed the highest CO_2_ production after 48 h (0.33 g/100 mL CO_2_).

**Figure 3:**
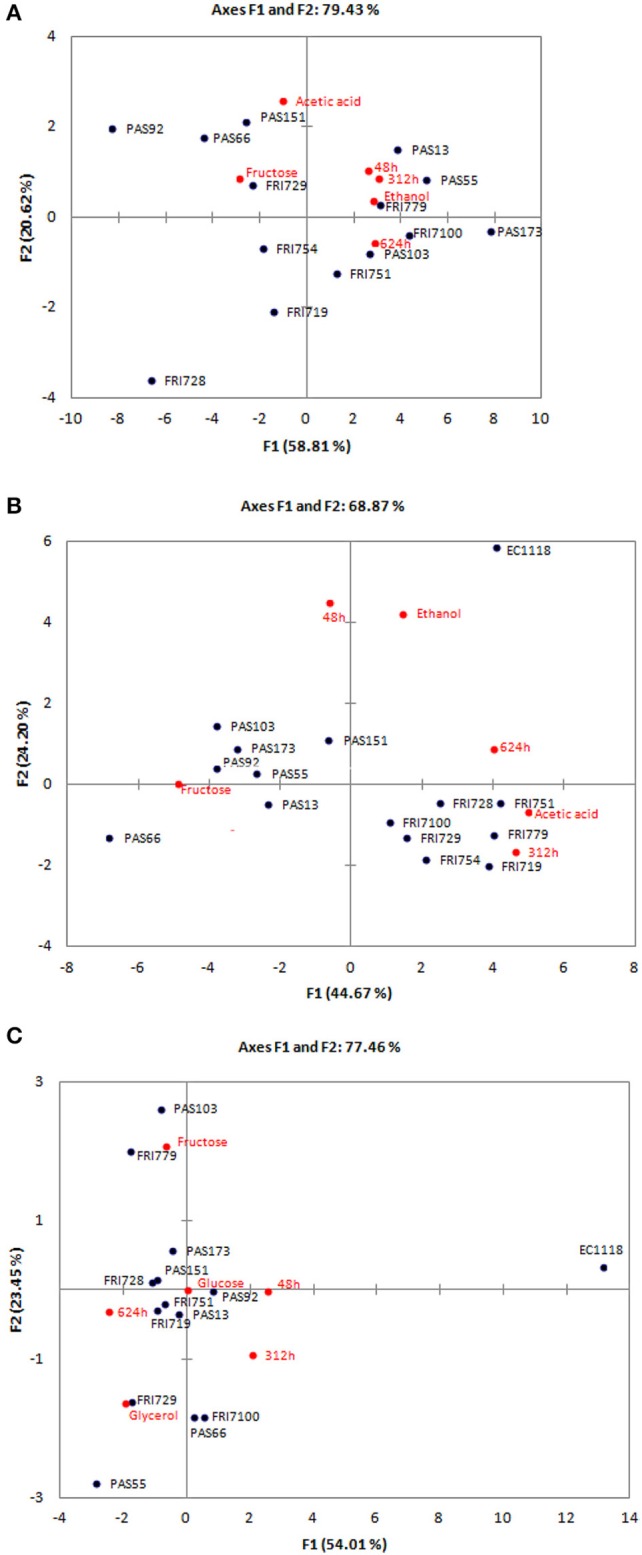
**Principal component analysis (PCA) biplot showing fermentation performances (CO_**2**_ production after 48, 312, and 624 h, glucose and fructose residues, ethanol, glicerol, and acetic acid production) of ***S. bacillaris*** strains in MS300 (A); in sequential fermentation with EC1118 in MS300 (B) and in sequential fermentation with EC1118 in natural must (C)**. Only variables that showed significant correlations are reported.

Concerning residual sugars, as expected, *S. bacillaris* consumed more fructose than glucose due to its fructophilic aptitude (Englezos et al., 2015). The sugars residues were very high (from 100.41 to 135.87 g/L), this was related to a limited ethanol production (from 4.12 to 6.19% v/v). Regarding secondary metabolites, their production was strongly strain dependent. As expected glicerol production was very high (from 5.58 to 7.81 g/L), while acetic acid concentration was generally limited (from 0.28 to 0.45 g/L). In order to evaluate differences in fermentation performances among the strains all the collected data (CO_2_ production after 48, 312, and 624 h, and the concentration of glucose and fructose residues, glicerol, acetic acid and ethanol) were analyzed by PCA (Figure 3A). Function (F1) accounted for 58.81% of the total variance and significantly correlated (α < 0.001) with CO_2_ production after 312, 624, and (α < 0.01) 48 h, with fructose residue and (α < 0.01) ethanol concentration. The second function (F2) explained 20.62% of the total variance and was correlated (α < 0.01) with acetic acid concentration. No significant correlations were found with glucose residue and glicerol production. The analysis confirmed the high level of similarity between the fermentation performances of the different strains when they are tested as single starter, irrespectively of the strain origin. FRI728 and PAS92 confirmed to be the strains with the worst fermentation aptitudes in terms of fermentation rate, and ethanol production. On the contrary PAS 173 showed the best fermentation performances.

All the strains were tested in co-fermentation with strain EC1118 in synthetic must MS300 at 20°C (see Supplementary Material SM_4). Each time sequential inoculations were performed adding *S. bacillaris* strain at first, followed, after 48 h, by *S. cerevisiae* EC1118. Both strains were added at the same concentration (1–1.5 × 10^6^ cells/ml). Strains PAS55, PAS66, PAS92, PAS103, PAS151, and PAS173 that showed high fermentation vigor (CO_2_ production after 48 h), evidenced a significant lower CO_2_ production after 312 h than that of the other strains. This means that after the addition of EC1118 a lower fermentation rate than that of EC1118 single-strain fermentation occurred. These strains, together with PAS13, showed the presence of sugar residues, although at low concentrations (from 1.92 to 7.9 g/L), confirming a lower fermentation rate than that of the other strains. The alcohol content was significantly higher in EC1118 single-strain fermentation than in sequential fermentations. Ethanol concentration in EC1118 single-strain fermentation was 13.16% (v/v), whereas sequential fermentations, where the reducing sugar were completely consumed, produced an average of 12.15% (v/v) ethanol, reducing 1% the alcohol content. These results confirmed the well-known ability of *S. bacillaris* species to reduce alcohol content in wine (Bely et al., 2013). Glicerol concentration was significantly lower in EC1118 single-strain fermentation (5.77 g/L) than in co-fermentations (average value 7.05 g/L). An average increase of 1.28 g/l was found. Strain glicerol production seems not to be related to sugar consumption, as strain PAS 13, that left 3.66 g/L of sugars, showed one of the highest levels of glicerol production. Only small differences were found in acetic acid production.

All the data obtained were analyzed by PCA (Figure 3B). Function (F1) accounted for 44.67% of the total variance and significantly correlated (α < 0.001) CO_2_ production after 312 h and (α < 0.05) 624 h, with fructose residue and acetic acid production concentration. The second function (F2) explained 24.20% of the total variance and was correlated (α < 0.01) with fermentation vigor and ethanol production. No significant correlations were found with glucose residue and glicerol production. In these conditions, strain origin seems to be the explanation of the strain clustering. “PAS” strains isolated from grape must B showed the worst fermentation performances with the presence of fructose residues, whereas “FRI” strains showed good fermentation performances, producing the highest level of acetic acid. EC1118 single strain fermentation clustered separately due to higher fermentation vigor and ethanol production than the sequential fermentations.

### Fermentation activity in natural must

Sequential fermentations of *S. bacillaris* and *S. cerevisiae* EC1118 were run in natural must in the same condition used for the synthetic must (see Supplementary Material SM_5). In this case the widest range of CO_2_ production was found after 288 h. EC1118 completed the fermentation within 12 days, sequential fermentations in 16 days. Only when FRI754, FRI779 and PAS103 were tested a limited fructose residue was found (from 1.47 to 2.40 g1/L). Glicerol was significantly higher in sequential fermentations than in EC1118 single strain fermentation. Glicerol concentration in EC1118 single strain fermentation was 4.86 g/l, whereas sequential fermentations produced an average of 5.84 g/L glicerol, with an average increase of 0.98 g/L. Ethanol concentration ranged between 11.19 and 11.61% (v/v). No significant differences were found between EC1118 fermentation and sequential fermentations for 5 out of 14 strains tested. This could be due to the lower sugar concentration present in the natural must than in the synthetic must of the previous trial. Indeed, glicerol production is directly proportional to the sugar content: the higher the sugar content, the higher the glicerol concentration, therefore the lower the ethanol concentration (Tilloy et al., 2014). Acetic acid concentrations were very limited and lower than those found during synthetic must fermentations (ranging from 0.28 to 0.36 g/L). All the data obtained were analyzed by PCA (Figure 3C). Function (F1) accounted for 54.01% of the total variance and significantly correlated (α < 0.001) CO_2_ production after 48, (α < 0.05) 312, and 624 h, and (α < 0.05) glicerol production. The second function (F2) explained 23.45% of the total variance and was correlated (α < 0.001) glucose and (α < 0.05) fructose residue. No significant correlations were found with acetic acid and ethanol content. In these conditions differences between sequential fermentations and EC1118 single-strain fermentation were more evident than in synthetic must in term of fermentation performances. In all the sequential fermentations a slower fermentation rate than that of EC1118 single-fermentation was found. The main differences among sequential fermentations were due to the presence of different level of sugar residues (ax F2).

## Discussion

With the aim of selecting wine yeasts carrying antifungal activity, fermenting musts obtained from late-harvest, overripe grape variety, naturally dried, were considered. The overripe grape berries show a very soft texture, due to the senescence or aging of fruit tissues. These physical features increase susceptibility to mechanical damage and infection by fungal pathogens (Genovese et al., 2007). Molds, such as *Botrytis cinerea* are abundant in this environment and yeasts must carry antifungal activity to compete. After yeast isolation and identification, 36 isolates were found to belong to the *S. bacillaris* species. This yeast possesses a fructophilic character and a poor ethanol yield from sugar consumed (Magyar and Tóth, 2011). Several ecological studies evidenced the presence of this species on grape berry surface and during spontaneous fermentations of musts in several countries (Bokulich et al., 2013a,b; Milanović et al., 2013; Wang et al., 2015), suggesting that this species has a specific role in the fermentation process. *S. bacillaris* carries some very interesting enological traits, such as growth at high concentrations of sugars and low temperatures (Sipiczki, 2003; Tofalo et al., 2012), and production of low levels of acetic acid, acetaldehyde and significant amounts of glicerol from consumed sugars (Magyar and Tóth, 2011). Contrary to the most common non-*Saccharomyces* yeasts, it can survive until the end of the alcoholic fermentation due to its ability to tolerate high concentrations of ethanol present in the wine (Rantsiou et al., 2012; Englezos et al., 2015).

By means of SAU-PCR analysis at least 14 genetically defined groups were found.

One isolate for each group was selected to test antagonistic activity to *Botrytis cinerea* both *in vitro* and i*n vivo*. The results of the antagonistic activity *in vitro* assay, obtained growing simultaneously the yeast strains together with the fungal mycelium, demonstrated that yeast isolates were able to limit the causal agent of gray mold disease and this seems not to be related to the acidic condition of the environment (PDA medium at pH 3.5). The values of the inhibition of the radial mycelium growth were comparable with those previously found for other antagonistic yeasts (Parafati et al., 2015). Due to the different growth rate of *S. bacillaris* and *B. cinerea* on PDA medium, where *S. bacillaris* can not found the optimal growth conditions, a 4 days pre-incubation of yeast strains was performed before testing the antifungal activity in the following *in vitro* assays.

Since several mechanisms have been reported to play a significant role in the biocontrol activity of antagonistic yeasts, in this study we evaluated the possible role of the main biocontrol modes of action, such as production of VOCs and cell wall-degrading enzymes, in controlling the *in vitro* growth of *B. cinerea*. Plate assays evaluating cell-wall degrading enzymes (pectinolitic, proteolytic and chitinolytic activities) indicated that pectinolitic activity was not present, chitinolytic activity was evident for all the strains although at low level, and only two strains FRI719 and FRI751 showed potential to produce proteolytic enzymes as they grew well on plates containing skin milk. The results regarding VOCs production were more promising. These compounds have been shown to have an antifungal effect and contribute to the biocontrol activity found in several yeast species, such as *Wickerhamomyces anomalus, Candida intermedia*, and *Sporidiobolus pararoseus* (Druvefors and Schnürer, 2005; Huang et al., 2011, 2012). In particular, more recently Hua et al. (2014) demonstrated that the biocontrol ability of *W. anomalus* can be attributed to the production of 2-phenylethanol, a secondary alcohol which affects spore germination, growth, toxin production, and gene expression in *Aspergillus flavus*.

It is well-known that volatile molecules, such as higher alcohols and esters are produced by non-*Saccharomyces* wine yeasts and their concentration is strain dependent (Rojas et al., 2001; Clemente-Jimenez et al., 2004; Jolly et al., 2006, 2014).

The inhibition percentage of the radial mycelial growth, during *in vitro* plate assay, was very high indicating a strong antifugal activity and suggesting VOCs as main responsible for *S. bacillaris* antifungal effects. The inhibitory effect of the *S. bacillars* strains was further proven on wounded grape berries artificially inoculated with *B. cinerea*. All the strains were able in reducing *B. cinerea* gray mold decay. In particular the lesion diameter reduction was comparable with that found previously for other antifungal yeasts (Parafati et al., 2015). Regarding the ability of the yeast strains to survive and multiply in artificial wounds made on grapes, results indicated that after 5 days from the inoculation, after squeezing the berries, the cell concentrations were very high (from 2 to 3 × 10^6^ CFU/ml). This finding suggested that *S. bacillaris* strains can easily growth in the wound environment on grape berries and have a considerable colonizing potential. Due to the promising *S. bacillaris* antifungal activity and the well-proved enological property of this species, fermentation ability of the *S. bacillaris* strains isolated in this study were tested using an inoculum carrying a cell concentration similar to that found in the infected berries. This concentration is interesting from an enological point of view as natural yeast population size in grape must after pressing, usually ranges from 10^4^ to 10^6^ cells/ml (Fleet et al., 1984; Combina et al., 2005; Jolly et al., 2006). Moreover, in several studies where *S. bacillaris* was used in sequential fermentation together with *S. cerevisiae* the inoculum size was 10^6^ cell/ml and at this concentration this yeast produced positive effect on wine (Andorrà et al., 2010; Rantsiou et al., 2012). *S. bacillaris* single-strain fermentation confirmed the fructofilic character, the high glicerol production and a fermentation rate slower than that of *S. cerevisiae* EC1118 (Magyar and Tóth, 2011; Englezos et al., 2015). When sequential fermentations were performed in synthetic must *S. bacillaris* strains significantly increased glicerol content and reduced ethanol concentration. In sequential fermentations of natural must the mixed starters consumed all the reducing sugars (only in few cases a minimal sugar residues remained in the wine) and *S. bacillaris* significantly increased the glicerol content, although the fermentation rate was slower than that of EC1118 single-strain fermentation. In all the fermentation trial *S. bacillaris* strains produced very low acetic acid concentrations. The level is lower than that found for other *S. bacillaris* strains isolated from another Italian winemaking region (Englezos et al., 2015). This finding is very interesting as one of the main concerns in the use of non-*Saccharomyces* strains in winemaking is their propensity to produce high level of volatile acidity (Jolly et al., 2006).

In this paper we demonstrated for the first time that strains of *S. bacillaris* carry antifungal activity and this property can be used to control the growth of the fungal pathogen *B. cinerea* on grape. Moreover, the interesting enological properties possessed by these strains have been proven to enhance wine quality. The high wound colonization ability of *S. bacillaris* found in this work together with its propensity to colonize the grape berry surface (Wang et al., 2015) suggests that the use of this yeast as biocontrol agent on grape plant and berries could influence the following must fermentation, although the presence of *S. cerevisiae* is needed to complete the fermentation. Further studies will be needed to assess the efficacy of *S. bacillaris* as biocontrol agent directly in vineyard to couple the antifungal activity with the enological properties of these strains. In this sense our results provide a new insight in the management of non-*Saccharomyces* yeast for winemaking and open new prospects to a more integrated strategy for increasing wine quality.

## Author contributions

Principal investigator: WL. Designed research: WL, BB, CV, AG. Performed antifungal activity: WL, FF, CN. Performed genetic characterization: WL, BB, GC. Performed fermentation trials and HPLC analyses: WL, CN, MC. Wrote the manuscript: VC. Edited the manuscript: VC, AG, WL, BB, CN.

## Funding

The research financial support was provided by CAPES-Coordenação de Aperfeiçoamento de Pessoal de Nível Superior and MIUR Ex 60% (Ministero dell'Istruzione, dell'Università e della Ricerca) (Grant No. 60A08/3022/15).

### Conflict of interest statement

The authors declare that the research was conducted in the absence of any commercial or financial relationships that could be construed as a potential conflict of interest.
