# Corrigendum: Biocontrol Ability and Action Mechanism of *Starmerella bacillaris* (Synonym *Candida zemplinina*) Isolated from Wine Musts against Gray Mold Disease Agent *Botrytis cinerea* on Grape and Their Effects on Alcoholic Fermentation

**DOI:** 10.3389/fmicb.2016.01499

**Published:** 2016-09-27

**Authors:** Wilson José Fernandes Lemos Junior, Barbara Bovo, Chiara Nadai, Giulia Crosato, Milena Carlot, Francesco Favaron, Alessio Giacomini, Viviana Corich

**Affiliations:** ^1^Department of Agronomy Food Natural Resources Animals and Environment, University of PadovaLegnaro, Italy; ^2^Interdepartmental Centre for Research in Viticulture and Enology, University of PadovaConegliano, Italy; ^3^Department of Land Environment Agriculture and Forestry, University of PadovaLegnaro, Italy

**Keywords:** antifungal activity, SAU-PCR, grape must, VOCs, lytic enzymes, fermentation, glicerol

Reason for Corrigendum:

Due to an incorrect earlier citation, the name of the first author should now read Wilson José Fernandes Lemos Junior.

The original article has been updated.

## Conflict of interest statement

The authors declare that the research was conducted in the absence of any commercial or financial relationships that could be construed as a potential conflict of interest.

