# Supplementary material for: Biocontrol Ability and Action Mechanism of Starmerella bacillaris (Synonym Candida zemplinina) Isolated from Wine Musts against Gray Mold Disease Agent Botrytis cinerea on Grape and Their Effects on Alcoholic Fermentation
Source: Front Microbiol. 2016 Aug 15;7:1249. doi: 10.3389/fmicb.2016.01249 (PMC4983571; doi:10.3389/fmicb.2016.01249)
Supplement: Supplementary file 1 [file Table1.doc]

***Supplementary Material***

**Biocontrol ability and action mechanism of *Starmerella bacillaris* (synonym *Candida zemplinina*) isolated from wine musts against grey mold disease agent *Botrytis cinerea* on grape and their effects on alcoholic fermentation**

Wilson Josè Fernandes Lemos Junior^1^, Barbara Bovo^1,2^, Chiara Nadai^1,2^, Giulia Crosato^1,2^, Milena Carlot^1,2^, Francesco Favaron^3^, Alessio Giacomini^1,2,*^, Viviana Corich^1,2^

***Correspondence**: Alessio Giacomini: [alessio.giacomini@unipd.it](mailto:alessio.giacomini@unipd.it)

**Supplementary figures and tables**


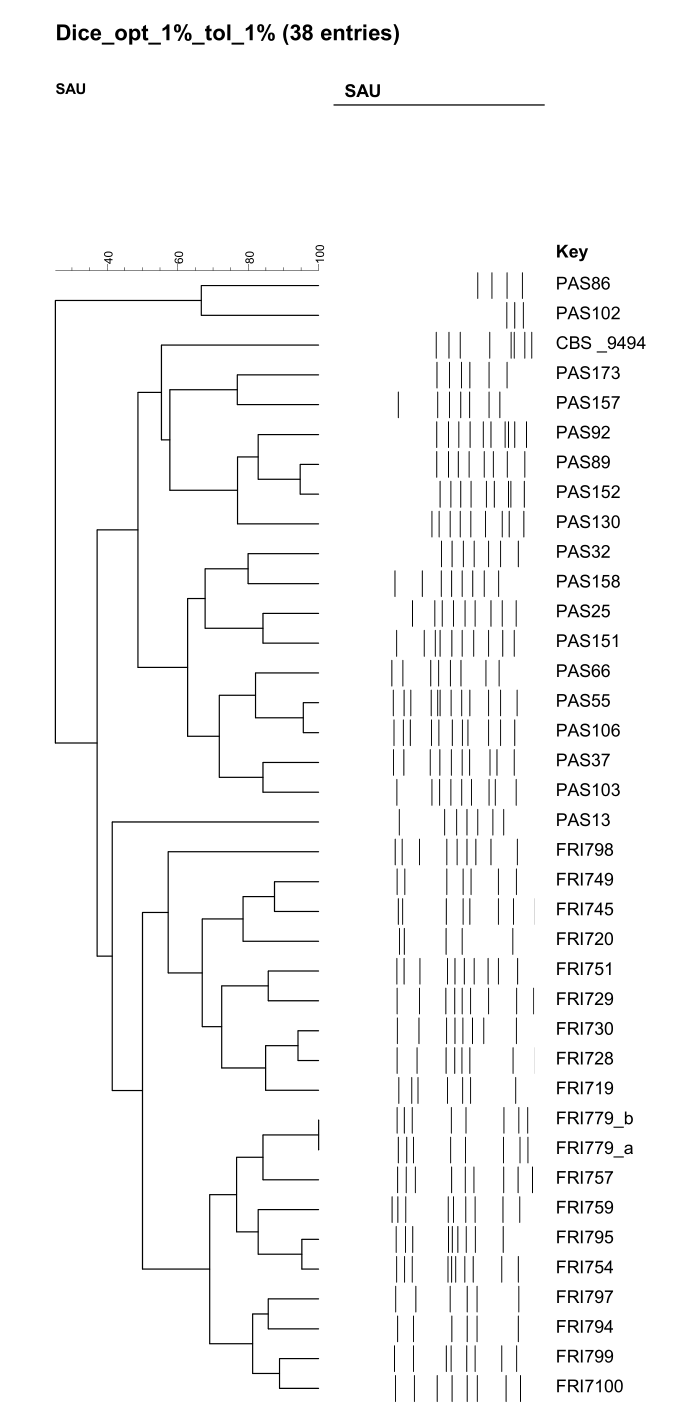
**SM_1.** Cluster analysis of Sau-PCR profiles obtained from 36 *S. bacillaris* isolates collected from Raboso Piave musts. FRI779_a and FRI799_b profiles were obtained by repeating Sau-PCR analysis twice on isolate FRI779. Strain CBS_ 9494 is a *S. bacillaris* type strain. The amplification patterns were analyzed using the software GelCompar II V. 3.5 (Applied Maths).

**SM_2.** Extracellular enzymatic activities of the 14 *S. bacillaris* strains isolated from Raboso piave musts.

| Strain | PrA | CellA | | XylA | | LipA | | *β-*gluA | | *β-*gluA | | PectA | | ChitA | |
| --- | --- | --- | --- | --- | --- | --- | --- | --- | --- | --- | --- | --- | --- | --- | --- |
|  | skinmilk | CMC | | Xylan | | tributim | | cellobiose | | esculin | | polygalacturonic acid | | glycolchitin | |
| FRI719 | + | | - | | - | | - | | - | | - | | - | | + |
| FRI728 | - | | - | | - | | - | | - | | - | | - | | + |
| FRI729 | - | | - | | - | | - | | - | | - | | - | | + |
| FRI751 | + | | - | | - | | - | | - | | - | | - | | +++ |
| FRI754 | - | | - | | - | | - | | - | | - | | - | | ++ |
| FRI779 | - | | - | | - | | - | | - | | - | | - | | ++ |
| FRI7100 | - | | - | | - | | - | | - | | - | | - | | + |
| PAS13 | - | | - | | - | | - | | - | | - | | - | | + |
| PAS55 | - | | - | | - | | - | | - | | - | | - | | + |
| PAS66 | - | | - | | - | | - | | - | | - | | - | | + |
| PAS92 | - | | - | | - | | - | | - | | - | | - | | + |
| PAS103 | - | | - | | - | | - | | - | | - | | - | | ++ |
| PAS151 | - | | - | | - | | - | | - | | - | | - | | ++ |
| PAS173 | - | | - | | - | | - | | - | | - | | - | | ++ |

PrA proteolytic activity, CellA cellulolytic activity, XylA xylan-degrading activity, LipA lipolytic activity, *β-*gluA *β-*glucosidase activity, PectA pectinolytic activity, ChitA chitinolytic activity; + very faint, ++ low, +++ medium, ++++ high.

**SM_3.** CO_2_ production during fermentation, residual glucose and fructose concentrations and concentrations of the main fermentation products at the end of the fermentation of *S. bacillaris* strains in MS300. Data are expressed as the average of three replicates ± standard deviations. Within the column, mean values followed by the same letter are not significantly different according to Fisher’s test (p ≤ 0.05).

| **Strain** |  | **CO_2_/100mL** |  | **Glucose (g/L)** | **Fructose (g/L)** | **Glycerol (g/L)** | **Acetic acid (g/L)** | **Ethanol (%v/v)** |
| --- | --- | --- | --- | --- | --- | --- | --- | --- |
|  | **48 h** | **312h** | **624h** |  |  |  |  |  |
| FRI719 | 0.03 ± 0.00^D^ | 1.58 ± 0.25^A^ | 3.63 ± 0.58^A^ | 85.32 ± 1.12^EF^ | 26.53 ± 1.14^EF^ | 5.58 ± 0.02^E^ | 0.39±0.09^ABCDE^ | 4.58 ± 0.01^E^ |
| FRI728 | 0.01 ± 0.00^D^ | 1.06 ± 0.02^A^ | 2.97 ± 0.10^A^ | 84.92 ± 3.10^EFG^ | 32.93 ± 1.19^C^ | 4.77 ± 0.30^F^ | 0.28±0.03^G^ | 4.15 ± 0.01^E^ |
| FRI729 | 0.22 ± 0.03^BC^ | 1.57 ± 0.12^A^ | 3.12 ± 0.23^A^ | 90.39 ± 0.35^CD^ | 29.07 ± 1.16^DE^ | 6.03 ± 0.15^E^ | 0.42±0.03^ABCD^ | 4.45 ± 0.02^E^ |
| FRI751 | 0.26 ± 0.04^AB^ | 1.81 ± 0.29^A^ | 3.28 ± 0.50^A^ | 78.22 ± 2.41^H^ | 24.49 ± 1.84^FG^ | 5.72 ± 0.20^E^ | 0.38±0.05^ABCDEF^ | 4.52 ± 0.024^E^ |
| FRI754 | 0.27 ± 0.02^BC^ | 1.64 ± 0.06^A^ | 3.10 ± 0.07^A^ | 91.5 ± 0.01^BCD^ | 31.51 ± 0.56^CD^ | 5.58 ± 0.60^E^ | 0.29±0.01^GF^ | 4.48 ± 0.01^E^ |
| FRI779 | 0.24 ± 0.02^BC^ | 1.8 ± 0.07^A^ | 3.60 ± 0.11^A^ | 89.75 ± 1.81^D^ | 21.01 ± 0.38^I^ | 7.05 ± 0.12^BC^ | 0.34±0.02^DEFG^ | 5.12 ± 0.02^E^ |
| FRI7100 | 0.27 ± 0.04^AB^ | 2.00 ± 0.27^A^ | 3.74 ± 0.50^A^ | 82.87 ± 0.64^FG^ | 25.07 ± 0.19^FG^ | 7.05 ± 0.04^BC^ | 0.32±0.03^GF^ | 4.86 ± 0.01^C^ |
| PAS13 | 0.28 ± 0.04^AB^ | 1.93 ± 0.19^A^ | 3.54 ± 0.33^A^ | 85.46 ± 2.10^E^ | 23.87 ± 1.31^GH^ | 7.81 ± 0.36^A^ | 0.40±0,02^ABCD^ | 4.94 ± 0.01^CD^ |
| PAS55 | 0.28 ± 0.04^AB^ | 1.99 ± 0.22^A^ | 3.82 ± 0.44^A^ | 86.33 ± 1.31^E^ | 21.25 ± 0.84^HI^ | 6.86 ± 0.53^BCD^ | 0.37±0.08^ABCDEF^ | 5.52 ± 0.03^B^ |
| PAS66 | 0.21 ± 0.02^BC^ | 1.46 ± 0.20^A^ | 2.93 ± 0.41^A^ | 93.26 ± 1.39^AB^ | 39.15 ± 2.14^B^ | 6.54 ± 0.31^D^ | 0.44±0.05^AB^ | 4.53 ± 0.02^E^ |
| PAS92 | 0.07 ± 0.02^D^ | 1.39 ± 0.26^A^ | 2.80 ± 0.43^A^ | 92.67 ± 0.28^ABC^ | 43.20 ± 0.05^A^ | 5.70 ± 0.01^E^ | 0.45±0.11^A^ | 4.12 ± 0.01^E^ |
| PAS103 | 0.26 ± 0.01^AB^ | 1.75 ± 0.01^A^ | 3.99 ± 0.95^A^ | 82.44 ± 0.27^G^ | 28.43 ± 1.26^GH^ | 6.63 ± 0.25^E^ | 0.35±0.05^BCDEFG^ | 4.55 ± 0.01^E^ |
| PAS151 | 0.16 ± 0.08^BC^ | 1.64 ± 0.11^A^ | 3.15 ± 0.20^A^ | 94.41 ± 1.63^A^ | 33.84 ± 4.01^C^ | 7.22 ± 0.19^B^ | 0.43±0.04^ABC^ | 4.49 ± 0.02^E^ |
| PAS173 | 0.33 ± 0.12^A^ | 2.05 ± 0.44^A^ | 3.77 ± 0.64^A^ | 78.63 ± 1.06^H^ | 21.78 ± 1.59^HI^ | 6.95 ± 0.16^BCD^ | 0.35±0.05^CDEFG^ | 6.19 ± 0.03^A^ |

**SM_4.** CO_2_ production during fermentation, residual glucose and fructose concentrations and concentrations of the main fermentation products at the end of the sequential fermentation of *S. bacillaris* strains with EC1118 in MS300. Data are expressed as the average of three replicates ± standard deviations. Within the column, mean values followed by the same letter are not significantly different according to Fisher’s test (p ≤ 0.05).

| **Strain** | **CO_2_/100mL** | | | **Glucose (g/L)** | **Fructose (g/L)** | **Glycerol (g/L)** | **Acetic acid (g/L)** | **Ethanol (%v/v)** |
| --- | --- | --- | --- | --- | --- | --- | --- | --- |
|  | **48 h** | **312h** | **624h** |  |  |  |  |  |
| EC1118 | 1.10 ± 0.14^A^ | 7.63 ± 0.34^B^ | 9.57 ± 0.20^A^ | - | - | 5.77±0.14^E^ | 0.51±0.02^A^ | 13.16±0.02^A^ |
| FRI719 | 0.01 ± 0.00^E^ | 8.66 ± 0.16^A^ | 9.38 ± 0.10^AB^ | - | - | 6.79±0.06^C^ | 0.52±0.01^A^ | 11.67±0.18^I^ |
| FRI728 | 0.01 ± 0.00^E^ | 7.21 ± 0.26^BC^ | 9.13 ± 0.06^BC^ | - | - | 6.69±0.01^C^ | 0.48±0.01^BC^ | 12.21±0.04^FG^ |
| FRI729 | 0.15 ± 0.03^DC^ | 7.57 ± 0.01^BC^ | 8.73 ± 0.11^DE^ | - | - | 7.47±0.19^B^ | 0.46±0.01^CDE^ | 12.19±0.06^FGH^ |
| FRI751 | 0.01 ± 0.01^E^ | 8.59 ± 0.21^A^ | 9.29 ± 0.18^AB^ | - | - | 6.57±0.29^C^ | 0.47±0.03^CD^ | 12.39±0.06^CDE^ |
| FRI754 | 0.26 ± 0.01^CB^ | 8.56 ± 0.04^A^ | 8.72 ± 0.12^DE^ | - | - | 8.26±0.30^A^ | 0.45±0.04^DEF^ | 12.22±0.10^EFG^ |
| FRI779 | 0.01 ± 0.00^E^ | 8.60 ± 0.33^A^ | 9.38 ± 0.07^AB^ | - | - | 6.82±0.13^C^ | 0.51±0.03^AB^ | 12.07±0.05^GH^ |
| FRI7100 | 0.13 ± 0.02^D^ | 6.94 ± 0.16^C^ | 8.73 ± 0.06^DE^ | - | - | 7.40±0.07^B^ | 0.44±0.01^EF^ | 12.28±0.02^DEF^ |
| PAS13 | 0.33 ± 0.06^B^ | 6.90 ± 0.97^C^ | 8.95 ± 0.05^CD^ | 1.36±0.68^B^ | 2.30±1.03^B^ | 7.59±0.14^B^ | 0.42±0.01^GF^ | 12.20±0.08^FG^ |
| PAS55 | 0.31 ± 0.07^B^ | 5.83 ± 0.63^D^ | 8.69 ± 0.17^DE^ | 0.12±0.22^C^ | 1.80±0.43^B^ | 6.85±0.23^C^ | 0.36±0.06^I^ | 12.02±0.28^H^ |
| PAS66 | 0.33 ± 0.09^B^ | 5.98 ± 0.91^D^ | 8.78 ± 0.39^DE^ | 3.14±0.35^A^ | 4.76±0.46^A^ | 6.67±0.11^C^ | 0.36±0.01^I^ | 11.42±0.01^J^ |
| PAS92 | 0.34 ± 0.04^B^ | 5.62 ± 0.27^DE^ | 8.64 ± 0.04^EF^ | 0.67±0.67^C^ | 2.58±1.25^B^ | 6.7±0.09^C^ | 0.36±0.01^I^ | 12.03±0.02^H^ |
| PAS103 | 0.28 ± 0.10^B^ | 5.01 ± 0.04^E^ | 8.38 ± 0.05^F^ | - | 2.49±0.23^B^ | 6.64±0.25^C^ | 0.38±0.02^HI^ | 12.55±0.02^C^ |
| PAS151 | 0.33 ± 0.06^B^ | 6.05 ± 0.19^D^ | 8.79 ± 0.06^DE^ | - | 0.08±0.14^C^ | 7.43±0.17^B^ | 0.40±0.02^GH^ | 12.75±0.04^B^ |
| PAS173 | 0.23 ± 0.01^BCD^ | 5.36 ± 0.42^DE^ | 8.60 ± 0.29^EF^ | - | 2.62±0.37^B^ | 6.85±0.03^C^ | 0.38±0.06^HI^ | 12.43±0.06^DC^ |

**SM_5.** CO2 production during fermentation, residual glucose and fructose concentrations and concentrations of the main fermentation products at the end of the sequential fermentation of *S. bacillaris* strains with EC1118 in natural must. Data are expressed as the average of three replicates ± standard deviations. Within the column, mean values followed by the same letter are not significantly different according to Fisher’s test (p ≤ 0.05).

| **Strain** | **CO_2_/100mL** | | | **Glucose (g/L)** | **Fructose (g/L)** | **Glycerol (g/L)** | **Acetic acid (g/L)** | **Ethanol (%v/v)** |
| --- | --- | --- | --- | --- | --- | --- | --- | --- |
|  | **48 h** | **288h** | **384h** |  |  |  |  |  |
| EC1118 | 2.17±0.07^A^ | 8.26±0.05^A^ | - | - | - | 4.86±0.06^D^ | 0.36±0.01^A^ | 11.61±011^A^ |
| FRI719 | 0.12±0.02^D^ | 6.29±0.21^DE^ | 7.78±0.11^CDE^ | - | - | 5.76±0.18^BC^ | 0.31±0.01^B^ | 11.34±0.21^BCDE^ |
| FRI728 | 0.14±0.02^CD^ | 5.70±0.10^GH^ | 7.39±0.02^GH^ | - | - | 5.82±0.17^BC^ | 0.32±0.01^B^ | 11.33±0.19^BCDE^ |
| FRI729 | 0.15±0.06^CD^ | 6.22±0.13^DEF^ | 7.77±0.16^CDE^ | - | - | 6.06±0.57^B^ | 0.29±0.02^B^ | 11.43±0.15^ABCD^ |
| FRI751 | 0.11±0.01^D^ | 6.96±0.09^C^ | 7.59±0.26^EFG^ | - | - | 5.79±0.47^BC^ | 0.31±0.02^B^ | 11.21±0.30^DE^ |
| FRI754 | 0.13±0.02^D^ | 5.66±0.15^H^ | 7.28±0.010^H^ | - | 2.05±0.42^B^ | 5.66±0.17^BC^ | 0.31±0.03^B^ | 11.19±0.14^E^ |
| FRI779 | 0.14±0.03^D^ | 6.1±0.47^DEFG^ | 7.68±0.28^DEF^ | - | 2.40±0.27^A^ | 5.77±0.25^BC^ | 0.31±0.05^B^ | 11.30±0.06^CDE^ |
| FRI7100 | 0.21±0.02^C^ | 7.02±0.16^C^ | 8.03±0.03^BC^ | - | - | 6.05±0.13^BC^ | 0.32±0.03^AB^ | 11.55±0.08^AB^ |
| PAS13 | 0.11±0.01^D^ | 7.56±0.08^B^ | 8.66±0.25^A^ | - | - | 5.80±0.20^BC^ | 0.32±0.02^B^ | 11.29±0.11^CDE^ |
| PAS55 | 0.35±0.04^B^ | 6.27±0.26^DEF^ | 7.92±0.10^BCD^ | - | - | 6.64±0.54^A^ | 0.28±0.03^B^ | 11.48±0.05^ABC^ |
| PAS66 | 0.13±0.01^D^ | 6.90±0.20^C^ | 7.49±0.3^FGH^ | - | - | 5.86±0.18^BC^ | 0.30±0.00^B^ | 11.53±0.04^AB^ |
| PAS92 | 0.15±0.06^CD^ | 6.33±0.16^D^ | 7.99±0.16^BC^ | - | - | 5.79±0.25^BC^ | 0.35±0.02^AB^ | 11.44±0.03^ABCD^ |
| PAS103 | 0.13±0.06^D^ | 5.88±0.11^FGH^ | 7.51±0.02^EFGH^ | - | 1.47±0.35^C^ | 5.46±0.07^C^ | 0.33±0.03^B^ | 11.25±0.07^DE^ |
| PAS151 | 0.13±0.06^D^ | 5.96±0.61^DEFG^ | 8.20±0.05^B^ | - | - | 5.69±0.25^BC^ | 0.32±0.01^B^ | 11.39±0.03^BCDE^ |
| PAS173 | 0.11±0.04^D^ | 5.91±0.04^EFGH^ | 8.12±0.11^B^ | - | - | 5.58±0.17^BC^ | 0.33±0.03^B^ | 11.35±0.14^BCDE^ |
